# Repurposing auranofin and meclofenamic acid as energy-metabolism inhibitors and anti-cancer drugs

**DOI:** 10.1371/journal.pone.0309331

**Published:** 2024-09-17

**Authors:** Sara Rodríguez-Enríquez, Diana Xochiquetzal Robledo-Cadena, Silvia Cecilia Pacheco-Velázquez, Jorge Luis Vargas-Navarro, Joaquín Alberto Padilla-Flores, Tuuli Kaambre, Rafael Moreno-Sánchez

**Affiliations:** 1 Laboratorio de Control Metabólico, Carrera de Médico Cirujano de la Facultad de Estudios Superiores Iztacala, Universidad Nacional Autónoma de México, Tlalnepantla, México; 2 Departamento de Bioquímica, Instituto Nacional de Cardiología Ignacio Chávez, Ciudad de México, México; 3 Center for Preventive Cardiology, Knight Cardiovascular Institute, Oregon Health & Science University, Portland, Oregon, United States of America; 4 Laboratorio de Control Metabólico, Carrera de Biología de la Facultad de Estudios Superiores Iztacala, Universidad Nacional Autónoma de México, Tlalnepantla, México; 5 Laboratory of Chemical Biology, National Institute of Chemical Physics and Biophysics, Tallinn, Estonia; University of Mosul, IRAQ

## Abstract

**Objective:**

Cytotoxicity of the antirheumatic drug auranofin (Aur) and the non-steroidal anti-inflammatory drug meclofenamic acid (MA) on several cancer cell lines and isolated mitochondria was examined to assess whether these drugs behave as oxidative phosphorylation inhibitors.

**Methods:**

The effect of Aur or MA for 24 h was assayed on metastatic cancer and non-cancer cell proliferation, energy metabolism, mitophagy and metastasis; as well as on oxygen consumption rates of cancer and non-cancer mitochondria.

**Results:**

Aur doses in the low micromolar range were required to decrease proliferation of metastatic HeLa and MDA-MB-231 cells, whereas one or two orders of magnitude higher levels were required to affect proliferation of non-cancer cells. MA doses required to affect cancer cell growth were one order of magnitude higher than those of Aur. At the same doses, Aur impaired oxidative phosphorylation in isolated mitochondria and intact cells through mitophagy induction, as well as glycolysis. Consequently, cell migration and invasiveness were severely affected. The combination of Aur with very low cisplatin concentrations promoted that the effects on cellular functions were potentiated.

**Conclusion:**

Aur surges as a highly promising anticancer drug, suggesting that efforts to establish this drug in the clinical treatment protocols are warranted and worthy to undertake.

## Introduction

In the search for more effective and affordable anti-cancer drugs, some non-steroidal anti-inflammatory drugs (NSAIDs) have shown encouraging results on deterring cell proliferation of several cancer types [1–3]. In particular, celecoxib has shown anti-cancer effects with negligible effects on non-cancer control cells at therapeutically relevant doses [3–5]. Aspirin, another repurposed NSAID, also displays anti-cancer effects [6], but this drug and other NSAIDs carry well-characterized severe side-effects such as gastrointestinal discomfort, mucosal erosions/ulcerations and bleeding [7, 8], like canonical chemotherapies as well. To attenuate this apparently unavoidable associated burden of cancer chemotherapies, novel drugs with null side-effects are required to be developed or found by repurposing or alternatively, searching for effective synergistic drug combinations in which the respective required doses are lower and their side-effects become negligible.

One of the more consistent emerging metabolism-derived features of cancer cells is their greater oxidative microenvironment [2]. The intrinsic increased oxidative stress at which cancer cells are subjected requires increased antioxidant defense mechanisms either by over-expressing antioxidant enzymes and/or by depending more on the supply of antioxidant molecules. This may have been the basis for testing auranofin (Aur; ([1-(thio-κS)-β-D-glucopyranose-2, 3, 4, 6-tetraacetyl] (triethylphosphine)-gold) as an anti-cancer drug. Aur was prescribed for treating inflammatory arthritis including rheumatoid and juvenile arthritis [9], but it is currently discontinuous. It is presumably a specific inhibitor of cytosolic and mitochondrial thioredoxin reductases (TrxR), antioxidant enzymes which catalyze the NADPH-dependent reduction of the redox protein thioredoxin (Trx) [10, 11]. Aur anti-cancer activity has been observed on a wide variety of cancer types [12] and the results have led to Aur clinical trials (https://classic.clinicaltrials.gov/).

Treatment with Aur caused cell death and impaired the growth of triple negative breast cancer cells (TNBC) grown as spheroids in 3D culture. Further, Aur exerted a significant *in vivo* anti-tumor activity in multiple TNBC models including human MDA-MB-231 xenograft, and syngeneic 4T1.2 and patient-derived xenografts (PDX) models. Aur also significantly inhibited the invasion potential of TNBC cells *in vitro* and significantly inhibited lung metastasis in 4T1.2 syngeneic model *in vivo* by decreasing the expression of various epithelial-mesenchymal transition markers [13].

However, Aur inhibition of TrxR seems insufficient for inducing cancer cell death [14]. In this regard, it has been observed a generalized oxidation of proteins involved in cell proliferation/cell division/cell cycle and cell-cell adhesion/cytoskeleton structure induced by Aur [14]. Thus, the Aur anti-cancer mechanisms have not been fully characterized. In addition to inhibition of TrxR 1 and 2 isoforms, Aur also targets the proteasome [15], nucleic acids and protein synthesis [16], signaling pathways [17–19], as well as glycolytic and mitochondrial proteins [2, 20–22]. Then, it appears interesting and relevant to systematically analyze the Aur effects on cancer energy metabolism.

Transcriptome profiling of lung cancer cell lines identified an Aur-resistance signature comprising 29 genes, most of which are targets of the transcription factor NRF2, such as those involved in glutathione metabolism and thioredoxin pathways. Hepatocellular carcinoma, non-small cell lung cancer, head-neck squamous cell carcinoma, and esophageal cancer carrying NFE2L2/KEAP1 mutations were predicted resistant, whereas leukemia, lymphoma, and multiple myeloma were predicted sensitive to Aur. The greater sensitivity of hematological cancers to Aur was further experimentally confirmed *i*.*e*., Aur-sensitive cancers show high dependence on glutathione and decreased expression of NRF2 target genes involved in GSH synthesis and recycling [23].

Meclofenamic acid (MA), another repurposed NSAID [24], has also shown anticancer effects on various cancer cell types [25] through different mechanisms including suppression of glycolysis, enhancement of mitochondrial activity, and interference with cellular networks. Upon MA treatment, small cell lung cancer cells exhibited suppression of glycolysis and enhancement of mitochondrial activity [26]. Proteomic analysis of prostate cancer cells revealed that proteins involved in glycolysis, cytoskeletal formation, transport activity, protein metabolism, and mRNA processing were notably affected by MA treatment [27]. In glioma cells, the fat mass and obesity-associated protein (FTO), an N^6^-methyladenosine (m^6^A) RNA demethylase, which regulates oncogene c-MYC activity, is inhibited by MA [28]. MA strongly interferes with the microtubule-mediated syncytial communicating intercellular network in glioblastoma [29].

Aur and MA have shown synergy with other drugs [12, 28]. For instance, combination of Aur and vitamin C efficiently killed human TNBC cells [30]. Buthionine sulfoximine that targets glutathione synthesis potentiated the Aur cytotoxicity and induced lethal oxidative stress in primary pancreatic cancer cells [31]. Aur in combination with the PARP inhibitor olaparib synergistically induced cytotoxicity in p53 mutant lung and pancreas cancer cells [32]. Similarly, MA enhanced the inhibitory effect of temozolomide on glioma cell proliferation [28]. MA also potentiated the apoptosis-induced gefitinib effect on non-small lung cancer cells by increasing gefitinib accumulation and decreasing FTO expression [33]. The combination of MA with simvastatin synergistically inhibited proliferation and migration of prostate cancer cells [34].

Thus, Aur and MA emerge as promising anti-cancer drugs with promiscuous multi-target activity. In order to assess whether the ATP producing pathways (glycolysis and oxidative phosphorylation) are a primary target of Aur or MA, a systematic analysis of energy metabolism including some ATP-dependent processes such as metastasis [35] was performed in intact cancer cells and isolated mitochondria. For comparative purposes, the effect of these drugs was also assayed in non-cancer cells, to establish whether the Aur and MA targeting is rather specific for cancer cells at the common doses used. Further research is required to understand the mechanisms of action of Aur and MA and to determine their specificity for cancer cells. Elucidating these mechanisms will contribute to the development of effective and safe treatment protocols for cancer patients.

## Material and methods

### Chemicals

Aur and MA (Sigma, MO, USA) were dissolved in a mix of 70% ethanol /30% dimethyl sulfoxide (DMSO). Ethanol/DMSO was less than 10% of the final volume in each culture dish and did not affect the proliferation rate and cellular viability in untreated control cells (>95%) and drug-treated cells (>90%).

### Animals

The experiments described with animals were approved by the Institutional Committee for the Care and Use of Laboratory Animals (Permit No. INC/CICUAL/002-2021). Disposal of biological residues was following the guidelines of the Mexican Official Norm (NOM-062-ZOO-1999). Female Wistar rats (200–250 g weight) were housed in the institution animal facility with 12h light-dark cycles and controlled temperature (18–26°C). The rats were fed with a standard pellet chow and water *ad libitum*.

### Cancer cell culture

All metastatic (breast MDA-MB231, MDA-MB468, cervix HeLa, prostate PC3, colorectal HCT116, COLO205, glioblastoma U373), low-metastatic (breast MCF-7) cancer and non-cancer (fibroblast 3T3, cardiomyocytes H9C2) cell lines (American Type Culture Collection, Rockville, MD, USA) used in this study were cultured in Petri dishes in 20 mL of Dulbecco’s Modified Eagle’s Medium (DMEM, Sigma, MO, USA) supplemented with 10% fetal bovine serum (Biowest, Mexico) and 10,000 U penicillin/streptomycin (Sigma-Aldrich, MO, USA). The genotyping (INMEGEN, México) of each cancer cell line revealed that the cells used shared more than 80% of the canonic allelic markers with the ATCC original clone. For growth and maintenance, cells were incubated in 5% CO_2_/95% air at 37°C and kept until 80–90% of confluence was reached. Then, cells were harvested, washed by centrifugation and resuspended in Krebs-Ringer buffer (KR, 125 mM NaCl, 5 mM KCl, 25 mM Hepes, 1 mM MgCl_2_, 1 mM KH_2_PO_4_, 1.4 mM CaCl_2_, pH 7.4) for further use [36]. For experimentation, cancer cells (3–5 x 10^6^ cells) were cultured in DMEM (with 25 mM glucose) for 24 h in the absence of drugs. Afterwards, old-medium was replaced with fresh DMEM medium (with 25 mM glucose) in the absence (control, non-treated cells) or in the presence of logarithmic concentrations of Aur and MA for 24 h more, under the same culture conditions described above. Viability of cultured cells was determined by the trypan blue exclusion assay [36]. The protein concentration was determined by the Lowry method [37].

### Analysis of Aur and cisplatin (CP) synergy by Bliss-type additivism

Cancer cells (3–5 x 10^6^ cells) were grown in DMEM for 24 h. Drugs (Aur and CP) were assayed at cell growth subIC_50_ (IC_50_ = concentration to reach 50% inhibition) doses under the same culture conditions described above. To identify synergy between Aur and CP, the mathematical model of Bliss-type additivism was applied [3]. This model describes the additive response “C” for two single drugs with effects A and B, following the equation: C = (A + B)—(A x B), where each effect is expressed as a fractional inhibition. To assess specificity of the drug toxicity, the Therapeutic Index Ratio (TI Ratio) was determined. TI ratio was calculated by dividing the IC_50_ values attained for non-cancer cells into the IC_50_ attained for cancer cells. A TI >3 indicates that the exposure to the drug results in low or negligible toxicity for normal cells and high toxicity for cancer cells [38].

### OxPhos and glycolysis fluxes in cancer cells

For glycolysis flux, cells (3 mg protein/mL) were incubated in KR buffer. Glycolysis was started by adding 5 mM external glucose (Sigma-Aldrich, MO, USA), and cellular samples were withdrawn after 0 and 10 min of incubation at 37°C under smooth orbital shaking. At the indicated times, the cells were rapidly mixed with 3% (w/v) ice-cold perchloric acid and centrifuged. The supernatants were neutralized with 1N KOH/100mM Tris. To rule out lactate production by glutaminolysis, cells were also incubated with 10 mM 2-deoxyglucose (Sigma-Aldrich, MO, USA). Lactate was determined by a standard method with lactate dehydrogenase (Roche, Mannheim, Germany) following the NADH formation at 340 nm [35].

For oxidative phosphorylation (OxPhos) flux, cells (2–5 mg protein/mL) were incubated at 37°C in air saturated KR medium *plus* 5 mM glucose. To distinguish the oxygen consumption associated to OxPhos, the cells were incubated with 5 μM oligomycin (Sigma-Aldrich, MO, USA), a potent, specific, and permeable inhibitor of the mitochondrial ATP synthase. The net OxPhos rate was determined by using a Clark type electrode, as previously described [36] and by using a high-resolution respirometer (Oroboros Instruments, Innsbruck, Austria) at 37°C. The contribution of OxPhos and glycolysis to the cellular ATP supply was determined, respectively, from the net OxPhos rate (oligomycin-sensitive respiration) multiplied by the ATP/O or P»/O_2_ ratio that corresponds to 2.5 or 5, and from the rate of lactate production, assuming a stoichiometry of 1 mol of ATP produced *per* 1 mol of lactate produced [35].

### Isolation of mitochondria from rat AS-30D hepatoma, heart and liver

AS-30D hepatocarcinoma cells were propagated in the peritoneal cavity of 200–250 g weight female Wistar rats by intra-peritoneal inoculation of 3 mL of a cellular suspension containing approximately 4 × 10^8^ cells/mL [36]. After 5–6 days, the rats were sacrificed by cervical dislocation and AS-30D ascites liquid was extracted from peritoneal area with a sterile syringe and collected in 50 mL-plastic tubes. The hepatoma cells were harvested by centrifugation and washed once by centrifugation in KR buffer. For mitochondria isolation from AS-30D hepatoma [39], cells were permeabilized with digitonin (Sigma Aldrich, CA, USA) at 10–40 μg/mg cellular protein concentration [40].

Rat liver (RLM) [41] and heart (RHM) [42] mitochondria were isolated as described previously by differential centrifugation.

### Determination of oxygen consumption in isolated non-cancer and cancer mitochondria

Respiration of AS-30D, liver and heart mitochondria (1 mg protein/mL) was assayed oxygraphically with a Clark-type O_2_ electrode in KME (120 mM KCl, 20 mM Mops, 1 mM EGTA) buffer pH 7.2 *plus* 2 mM KH_2_PO_4_, and different oxidizable substrates (as indicated in Results). For state 3 respiration, 600 nmol ADP were added [40]. Mitochondria were incubated at 37°C with Aur and MA for 30–60 s before substrates were added. At the end of each respiratory measurement, dithionite was added to get the chemical zero oxygen signal.

### Cell migration and invasiveness

The wound healing assay was performed for cell migration detection. In petri dishes with full DMEM, cancer cells (5 x 10^6^ cells/well) were cultured at 37°C under 95% air and 5% CO2 until 80–90% confluence was attained. Afterwards, cell culture was wounded with a 1 mL pipette tip, washed twice with PBS (155 mM NaCl, 1.5 mM KH_2_PO_4_, 2.7 mM NaH_2_PO_4_, pH 7.2) and subsequently incubated at 37°C with fresh non-serum DMEM. Cellular migration images were taken in an inverted microscope (Zeiss, Thornwood, NY, USA) after 0 and 24 h incubation time. Using a graduated reticule (Zeiss, Thornwood, NY, USA), cellular migration was assessed from the petri dish’s border to its center distance travelled [43].

The 96-multiwell Boyden chambers (Merck Millipore, MA, USA) assay was performed for cellular invasiveness detection. Cancer cells (5 × 10^4^ cells/well) were re-suspended in free-serum DMEM and placed in the upper Boyden chamber compartment. Free-serum DMEM was also placed in the lower compartment of the chamber. Afterwards, the Boyden chamber was placed at 37°C and 95% air/5% CO_2_ for 24 h. Invasive cancer cells within the lower chamber compartment were detected by using the calcein-AM (60 nM, 1 h) assay at 37°C. Calcein-fluorescence was measured at λexcitation = 485 nm and λemission = 520 nm in a Nunclon microplate reader (Roskilde, Denmark) [43].

### Determination of drug IC_50_ values in HeLa cells

For cell proliferation in the presence of drugs, cancer cells (20 x 10^3^ cells/well) were grown in 96-well plates containing DMEM (Sigma-Aldrich) *plus* 10% bovine fetal serum for 24 h. Afterwards, Aur or MA (0.01, 0.1, 10 and 100 μM) were added to the cell cultures and incubated for additional 24 h. The effect of these drugs on cell proliferation was determined in 96-well plates by the MTT (3-[4,5-dimethyl-thiazol-2-yl]-2,5-diphenyltetrazolium bromide) assay. The viability of cancer cells was > 95% after treatment with drugs.

### Mitophagy assay

The selective removal of damaged/inhibited mitochondria by autophagosomes and lysosomes (*i*.*e*., mitophagy) was detected by using an EVOS epifluorescence cell imaging microscope (ThermoFisher, Waltham MA, USA). HeLa cells (5 × 10^4^ cells/mL) were cultured in 2 mL DMEM in glass bottom culture dishes and exposed to Aur for 24 h. HeLa cells were incubated with 400 nM Hoechst (Bis-Benzimide H 33342 trihydrochloride), 500 nM MitoTracker-Green FM and 500 nM LysoTracker-Red for 30 min at 37°C for nuclei, mitochondria and lysosome detection, respectively. Afterwards, cells were washed, resuspended with DMEM medium without red phenol and placed in the EVOS microscope for their analysis.

### Statistical analysis

Data shown are mean ± standard deviation from at least three independent cell preparations (n). For statistical significance between experimental groups, Student’s *t* test and ANOVA/post hoc Scheffé [44, 45] analyses were used. Significance level was established at *p* ≤ 0.05 or lower.

## Results

### Potent auranofin inhibitory effect on human metastatic cancer cell proliferation

**Fig 1:**
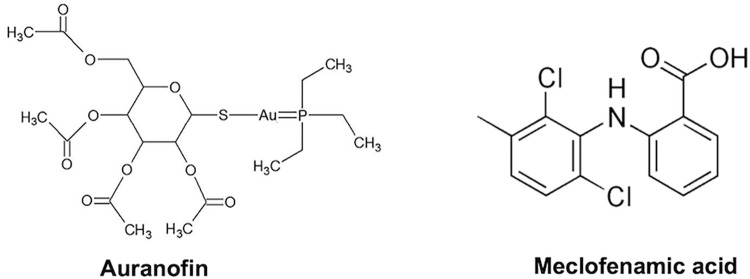
Auranofin and meclofenamic acid chemical structures.

To have a comparable rigorous assessment of the inhibition of cancer cell proliferation by anti-cancer drugs, several highly metastatic human cancer cell lines were assayed in parallel for their sensitivity to Aur or MA (Fig 1).

Under identical culture conditions and using the same methodology for determining cell proliferation, all metastatic cancer cell lines showed high sensitivity to Aur with IC_50_ values in the low micromolar range, including that for the frequently used low-metastatic breast cancer MCF-7 cell line (Table 1). On the other hand, the effects of MA were more varied (Table 1), inhibiting the growth of some cancer cells (HeLa, PC3) but not of others (HCT116, MCF-7). It is noted that cell viability estimated by 0.5% trypan blue exclusion was greater than 95% in untreated cells, whereas after 24 h drug exposure, it was higher than 90%. Exposure of HeLa cells to Aur or MA for shorter times (12 h) did not significantly affect proliferation (<15%).

**Table 1:** Sensitivity of cancer cell proliferation to auranofin (Aur) and meclofenamic acid (MA).

IC_50_ (μM) for proliferation cell rate		
Cells	Aur	MA
**Human metastatic cancer cells**		
Breast MDA-MB-231	0.8 ± 0.2 (3)	51 ± 5 (4)
Breast MDA-MB-468	2 ± 1 (3)	N.D.
Cervix HeLa	1.7 ± 0.4 (6)	6 ± 3 (4)
Prostate PC3	6.5 ± 3.5 (3)	6 ± 3 (3)
Colorectal HCT116	3 ± 1 (3)	>100 (3)
Colorectal COLO205	6 ± 2 (3)	N.D.
Glioblastoma U373	0.6 ± 0.1 (3)	N.D.
**Human low metastatic cancer cells**		
Breast MCF-7	5 ± 0.6 (3)	>100 (1)
**Non cancer cells**		
3T3 mouse fibroblasts	42 ± 17 (3)	>100 (3)
H9C2 mouse cardiomyocytes	78 ± 15 (3)	>100 (3)

The Aur and MA concentrations required to reach 50% inhibition of cell proliferation were determined from plots of drug concentration *versus* cell number or proliferation reached, after 24 h culture from initial inoculum of 3–5 x 10^6^, fitted by adjusting each value to a single decaying exponential function. Cell number was determined in a Neubauer chamber. The values shown represent the mean ± S.D., with the number of assayed cell preparation in parentheses. N.D., not determined.

Sulindac, another NSAID, was also tested for its effect on the same cancer cell lines displayed in Table 1. However, cancer cell proliferation was not significantly affected by up to 500 μM sulindac after 24 h incubation (S1 Table). Perhexiline, a carnitine palmitoyl transferase I and free fatty acid β-oxidation blocker, did show potent inhibitory effects on cancer cell proliferation with IC_50_ values in the 4–20 μM range (S1 Table), whereas its IC_50_ values for non-cancer cells were higher, in the 30–100 μM range. Simvastatin, a cholesterol (and quinones) synthesis blocker, exhibited a similar inhibition pattern on cancer cell proliferation to that displayed by perhexiline, with IC_50_ values of 31–147 μM for cancer cells and 100–222 μM for non-cancer cells (S1 Table). However, it was decided not to further pursue these other drugs in the present study and rather focus on Aur and MA.

To provide a comparative assessment, the inhibitory effects of Aur and MA on non-cancer cells (fibroblasts and cardiomyocytes) were also evaluated. These cells were nearly one order of magnitude less sensitive to Aur and MA than cancer cells. Analysis of the therapeutic index ratio (TI, S2 Table) revealed a TI >3 in all assayed conditions, except for MA in HCT-116, MCF-7 and MDA-MB-231, indicating that these drugs exerted low toxicity on normal cells and high toxicity on cancer cells [38]. Because of mouse non-cancer cells could not be the appropriated cell type for comparative purposes with the cancer cell lines used, drugs assays with other human non-cancer cells are underway. It is important to emphasize that in the majority of toxicological studies, the analysis of drug effects is not often performed on any type of non-cancer cells. Here, it is demonstrated the lesser effect of Aur and MA on healthy, normal non-cancer cells and in consequence, their potential use in clinical trials.

### Targeting cancer energy metabolism by Aur and MA

#### Effect on isolated cancer cells

Aur primary target is presumably TrxR. However, some studies have also shown effects of Aur on cancer energy metabolism, affecting both glycolysis and mitochondrial metabolism and function at the same doses that inhibit TrxR activity. Studies on MA effects on cancer energy metabolism are scarce and primarily have focused on the influence of MA on protein content analysis [26]. Therefore, to establish whether Aur and MA effectively affect energy metabolism in cancer cells at low micromolar concentrations, the sensitivity of energy metabolism pathway fluxes was assayed in intact living cancer cells (Table 2). In these assays, Aur and MA were added at their IC_50_ values determined for each particular cell line (Table 1).

**Table 2:** Inhibition of cancer energy metabolism by Aur and MA.

Metastatic cells	OxPhos			Glycolysis	
	nmol O_2_ min^-1^ mg protein^-1^			nmol lactate min^-1^ mg protein^-1^	
	Control	+Aur	+MA	Control	+Aur
HeLa	3.8 ± 1 (6)	2.6 ± 0.5*	1.1 ± 0.3*	16 ± 1 (3)	4 ± 3* (3)
MDA-MB-231	9.3 ± 0.6 (6)	2.5 ± 0.5*	3.1 ± 2*	11 ± 3 (3)	3 ± 2* (3)
MDA-MB-468	7.1 ± 0.8	N.D.	4.8 ± 0.8*	N.D.	N.D.

Aur and MA were added at their IC_50_ values taken from Table 1: 1.7 μM for HeLa cells, and 0.8 μM for MDA MB-231 cells. After 24 h, pathway fluxes were determined as described in Material and Methods section. Meclofenamic acid (MA) was added at 6 μM for HeLa cells but at 50 μM for MDA MB-231 and MDA-MB-468 cells. *P<0.01 vs. control (non-treated cells). N.D., not determined.

After 24 h incubation, Aur potently inhibited (48–75%) both oxidative phosphorylation (OxPhos) and glycolysis fluxes in HeLa and MDA-MB-231 cells (Table 2). MA also inhibited OxPhos flux by 31–66%, but at much higher concentrations than those used for Aur. Perhexiline at 10 μM inhibited by 36–70% oxygen consumption of HeLa, MDA MB-231, and A548 (lung cancer) cells. It should be noted that after 24 h incubation, 50% of the cells were dead, or they might be becoming functionally impaired, because drug doses used were at their IC_50_ values. Therefore, effects attained at this time-point could be related to cell death rather than to a specific drug effect on the function assessed. However, the following functional studies were performed with living cells, discarding dead cells.

#### Effect of Aur on respiration of isolated non-cancer and cancer mitochondria

Undoubtedly, it is more rigorous to compare metabolic functions and behavior of cervix and breast cells with those of their own isolated mitochondria. However, a large amount of cultured cancer cells is required to obtain an enriched mitochondrial fraction of high quality and purity. This goal cannot be achieved with human cancer cell cultures using the available commercial kits for preparing mitochondria. AS-30D hepatoma cells are a cancer cell model that allows for preparing functional, tightly coupled mitochondria with high yields [36, 40, 41, 46]. Thus, mitochondrial cancer metabolism and functions can be directly and reliably analyzed. For comparative purposes and as a control, respiration rates were also evaluated in isolated mitochondria from rat liver (RLM), which is the tissue of origin of AS-30D hepatoma. Then, in order to establish a direct, specific and general effect of Aur and MA in aerobic organs, these drugs were also assayed in isolated mitochondria from rat heart (RHM).

The direct interaction of Aur with mitochondria for short exposure times (3–7 min), in contrast to the long incubation times required for inhibiting cell proliferation, promoted a stimulation of pseudostate 4 (*i*.*e*., basal respiration in the absence of added nucleotides) and state 4 respiration (*i*.*e*., respiration rate attained after added ADP has been transformed into ATP) rates, indicating an uncoupling effect (Table 3 and S3 Table). A clear decrease of state 3 (*i*.*e*., ADP-stimulated respiration) and net state 3 (*i*.*e*., State 3 *minus* pseudo state 4) respiration rates indicated OxPhos inhibition by 100 μM Aur in both hepatoma and liver mitochondria with glutamate-malate (GM) (Table 3), or succinate (*plus* rotenone) as oxidizable substrate (S3 Table). Inhibitory effect on OxPhos by lower Aur concentrations was not so clear, probably because the short incubation times used. Interestingly, state 4 respiration rates driven by GM (Table 3) of hepatoma mitochondria showed a slightly greater sensitivity (2.3–2.4 times increase) to Aur than RLM (1.8–1.9 times increase). In turn, state 3 respiration rates in RLM displayed a slightly lower Aur sensitivity (20–54% inhibition) than in hepatoma mitochondria (42–75% inhibition). Likewise, state 4 and state 3 respiration rates driven by succinate (S3 Table) in RLM showed slightly lower sensitivity to Aur than hepatoma mitochondria. Hence, Aur did not show clear preference for cancer mitochondria. Then, the increased cholesterol content in the mitochondrial membranes [46, 47] and the slightly higher electrical membrane potential exhibited by cancer mitochondria [48] did not favor a specific anticancer effect, since normal mitochondria showed similar sensitivity towards Aur.

**Table 3:** Effect of Aur on oxygen consumption rates (nmol O_2_ min^-1^ mg protein^-1^) of cancer and non-cancer isolated mitochondria.

Hepatoma Mitochondria					
	0	10 μM	25 μM	50 μM	100 μM Aur
**5 mM Glutamate/ 0.1 mM Malate**					
**Pseudo State 4**	30 ± 5	33.5 ± 2	37 ± 5	52.5 ± 11*	67 ± 21.5*
**State 3**	155 ± 39	125 ± 9	114± 31	133± 46	125 ± 61
**State 4**	33.5 ± 11	44 ± 6	43 ± 3	74 ± 28*	79 ± 30*
**Net State 3 (State 3 –pseudoState 4)**	125 ± 34	91.5 ± 8	78 ± 29	80 ± 39	68 ± 41
**RC (State 3/ State 4)**	4.7 ± 0.7	2.8 ± 0.5	2.6 ± 0.5*	2.2 ± 0.5*	1.5 ± 0.4 *
**Rat Liver Mitochondria**					
	0	10 μM	25 μM	50 μM	100 μM Aur
**5 mM Glutamate/ 5 mM Malate**					
**Pseudo State 4**	26 ± 10	27.5 ± 3	27 (2)	30.5 (2)	48 (2)
**State 3**	126 ± 26	151 ± 61.5	89 (2)	78.5 (2)	73 (2)
**State 4**	14 ± 4	18 ± 5	15 (2)	16 (2)	26 (2)
**Net State 3**	100 ± 33	123.5 ± 59	62	48	25
**RC**	10 ± 5	8 ± 1	6 (2)	5.5 (2)	3 (2)

Isolated mitochondria (1 mg protein/mL) were incubated in KME buffer (120 mM KCl, 20 mM MOPS, 1 mM EGTA) pH 7.20 plus 2 mM KH_2_PO_4_ and the indicated oxidizable substrates. For state 3 (ADP-stimulated) respiration, 300–600 nmol ADP were added. RC, respiratory control; n = 3, P<0.01 *vs*. Control (no Aur added). To get the maximal reduction of molecular oxygen (zero oxygen concentration), sodium dithionite (Na_2_O_4_S_2_) was added at the end of each measurement.

Although a similar sensitivity pattern was observed for MA on hepatoma mitochondria as compared with liver and heart mitochondria (Table 4), it is noted that this pattern was achieved at one order of magnitude lower drug concentration in cancer mitochondria. The pseudostate 4 and state 4 respiration rates were stimulated by MA in hepatoma, liver and heart mitochondria at similar extents, whereas the state 3 and net state 3 respiration rates were also inhibited at similar extents. Thus, both NSAIDs showed no clear specificity for isolated cancer mitochondria over non-cancer mitochondria, which suggested that whether a specific inhibitory effect of Aur and MA on cancer cells does exist, this is not linked to their direct interaction with mitochondria. Therefore, the potential anticancer effects of these drugs may be related to their interaction with other cellular components in the cytosol or plasma membrane.

**Table 4:** Effect of MA on oxygen consumption rates of cancer and non-cancer isolated mitochondria.

Hepatoma Mitochondria			
	0	5 μM	10 μM MA
**5 mM Glutamate/ 0.1 mM Malate**			
**Pseudo State 4**	17±6 (3)	19 ± 4 (3)	45 ± 15* (3)
**State 3**	64 ± 7 (3)	45 ± 10* (3)	36 ± 7* (3)
**State 4**	18 ± 3 (3)	45 ± 10* (3)	36 ± 7* (3)
**Net State 3**	47 ± 5.5	26 ± 13	0
**RC (State 3/ State 4)**	3.6 ± 0.4 (3)	1 (3)	1 (3)
**Rat Liver Mitochondria**			
	0	10 μM	50 μM MA
**5 mM Glutamate/ 5 mM Malate**			
**Pseudo State 4**	26.5 ± 9 (3)	32.5 (1)	42 ± 15 (3)
**State 3**	103.5 ± 13 (3)	113.5 (1)	102 ± 23 (3)
**State 4**	11.5 ± 2.5 (3)	13.5 (1)	50 ± 17* (3)
**Net State 3**	77 ± 13	81 (1)	61 ± 27
**RC**	9.2 ± 1.4 (3)	8.6 (1)	2.1 ± 0.2* (3)
**Rat Heart Mitochondria**			
	0	10 μM	50 μM MA
**5 mM Glutamate/ 5 mM Malate**			
**Pseudo State 4**	55 ± 2 (3)	143 ± 42 (3)	134 ± 9.5 (3)
**State 3**	336 ± 12 (3)	177 ± 56* (3)	139 ± 22* (3)
**State 4**	83 ± 22 (3)	126 ± 16* (3)	139 ± 22* (3)
**Net State 3**	281 ± 14	48 ± 83	12.5± 22
**RC**	4.1 ± 0.9 (3)	1.4 ±0.6* (3)	1 (3)

### Aur induces mitophagy but not ROS production in cancer cells

**Fig 2:**
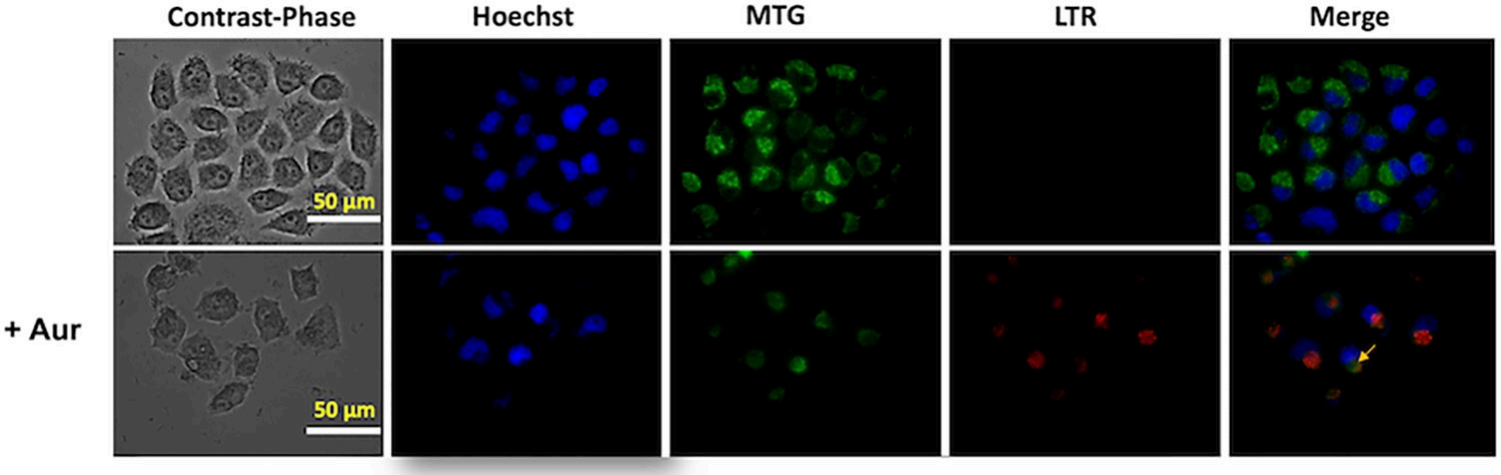
Auranofin (Aur) promotes cancer cell mitophagy in HeLa cells. Cells were exposed to IC_50_ Aur (1.7 μM) for 24 h. Cells were cultured in DMEM in glass-bottom culture dishes. Yellow arrows show the MTG-LTR co-loading. The figure shows a representative experiment, *i*.*e*. *n* = 1, 10–15 cells.

To establish which cell processes were associated to Aur-dependent OxPhos inhibition, mitochondrial digestion (mitophagy) and ROS production were assayed in HeLa cells (Fig 2). Mitophagy is the selective removal of dysfunctional or damaged mitochondria by autophagosomes, which is triggered by mitochondrial inner membrane depolarization and the generation of reactive oxygen species (ROS) [49]. Mitophagy was assessed by the number of lysosomes and their co-localization with mitochondria in LTR/MTG-loaded cells by epifluorescence microscopy. Staining of cancer cells with MitoTracker green (MTG), which covalently binds to the thiols of mitochondrial proteins and accumulates in the mitochondrial matrix regardless of the mitochondrial transmembrane electrical potential, revealed abundant and functional mitochondria. In contrast, cell staining with LysoTracker red (LTR), which accumulates inside organelles with internal acidic pH, showed scarce lysosomes. Then, it should be noted that MTG staining predominantly reveals living cells rather than dying cells. In addition, dead cells were previously removed for the dye loading procedure, thus discarding unspecific drug effects on mitophagy.

**Fig 3:**
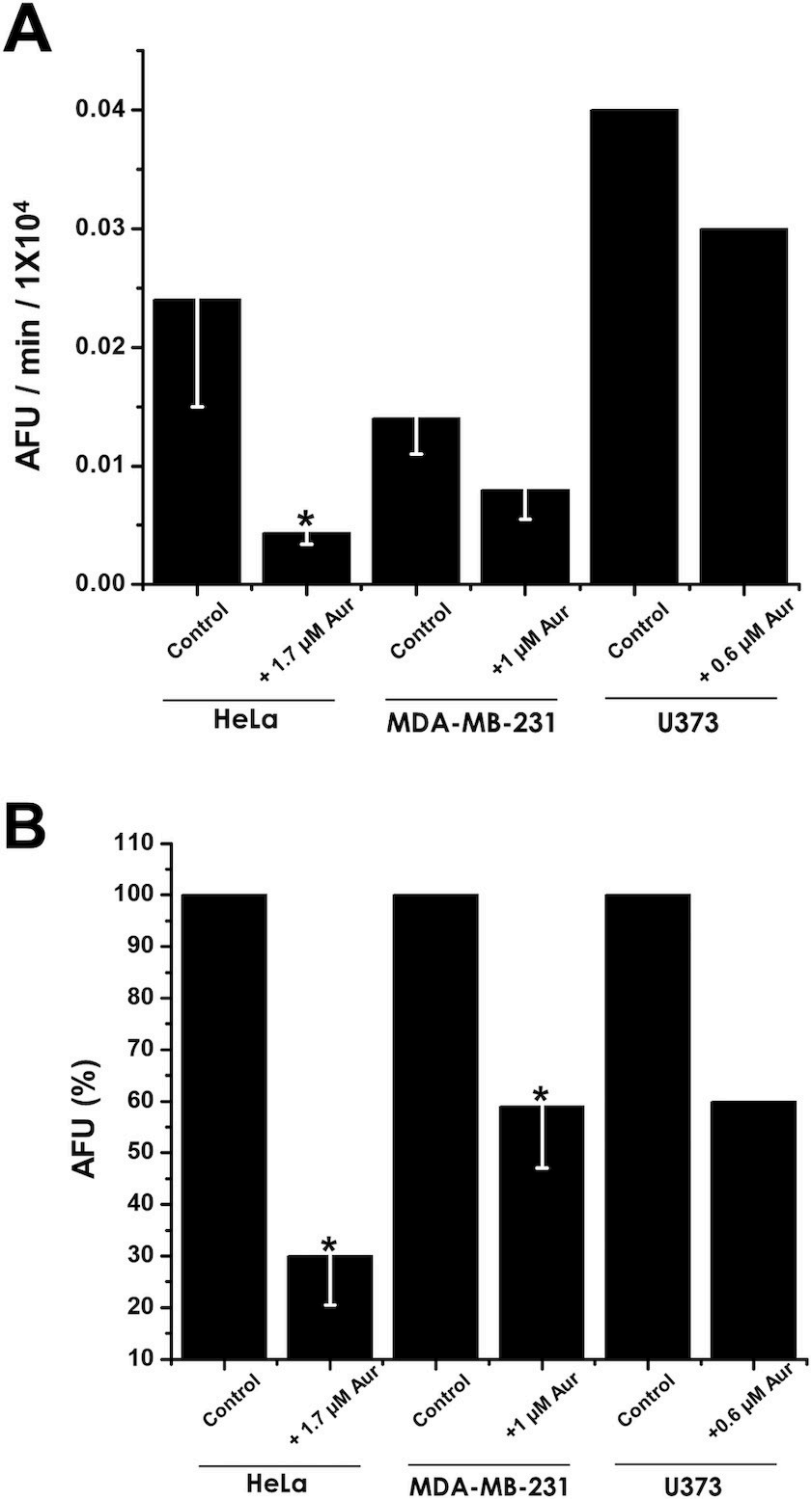
Radical oxygen species in Aur-treated cancer cells. (A) ROS production and (B) ROS total content in DHE loaded-metastatic cancer cells. The 100% fluorescence corresponds to 1.9 ± 0.26 (n = 3); 1.2 ± 0.09 (n = 3); and 0.64 (n = 2) fluorescence arbitrary units for HeLa, MDA-MB-231 and U373 cells, respectively. The Aur concentration added was 1.7, 0.8 and 0.6 μM for HeLa, MDA-MB-231 and U373 cells, respectively. The contents of ROS shown were determined at the 120 min end-point, after adding 25 μM DHE. *p < 0.05 vs. Control (Non- treated cells).

Incubation with Aur prompted a marked decrease in the mitochondrial signal in HeLa cells and a moderate increment in lysosomal signal indicating a severe loss in mitochondrial number/function (Fig 2). Co-localization of MTG mitochondria and LTR lysosomes (yellow arrows) in HeLa cells treated with Aur, indicated a significant enhancement in the mitophagy process. As a consequence of OxPhos inhibition, higher intracellular ROS production was expected [2]. However, exposing HeLa cells for 24 h to IC_50_ Aur concentration (1.7 μM) did not bring about increments in ROS production rate (Fig 3A) and total ROS levels after 2 h measurement (Fig 3B). These results show that Aur treatment induces mitophagy and impairs mitochondrial function in cancer cells, but an increased ROS production did not occur in HeLa cells under the conditions tested, probably derived from its uncoupling effect on mitochondrial respiration.

### Metastasis is decreased by Aur

**Fig 4:**
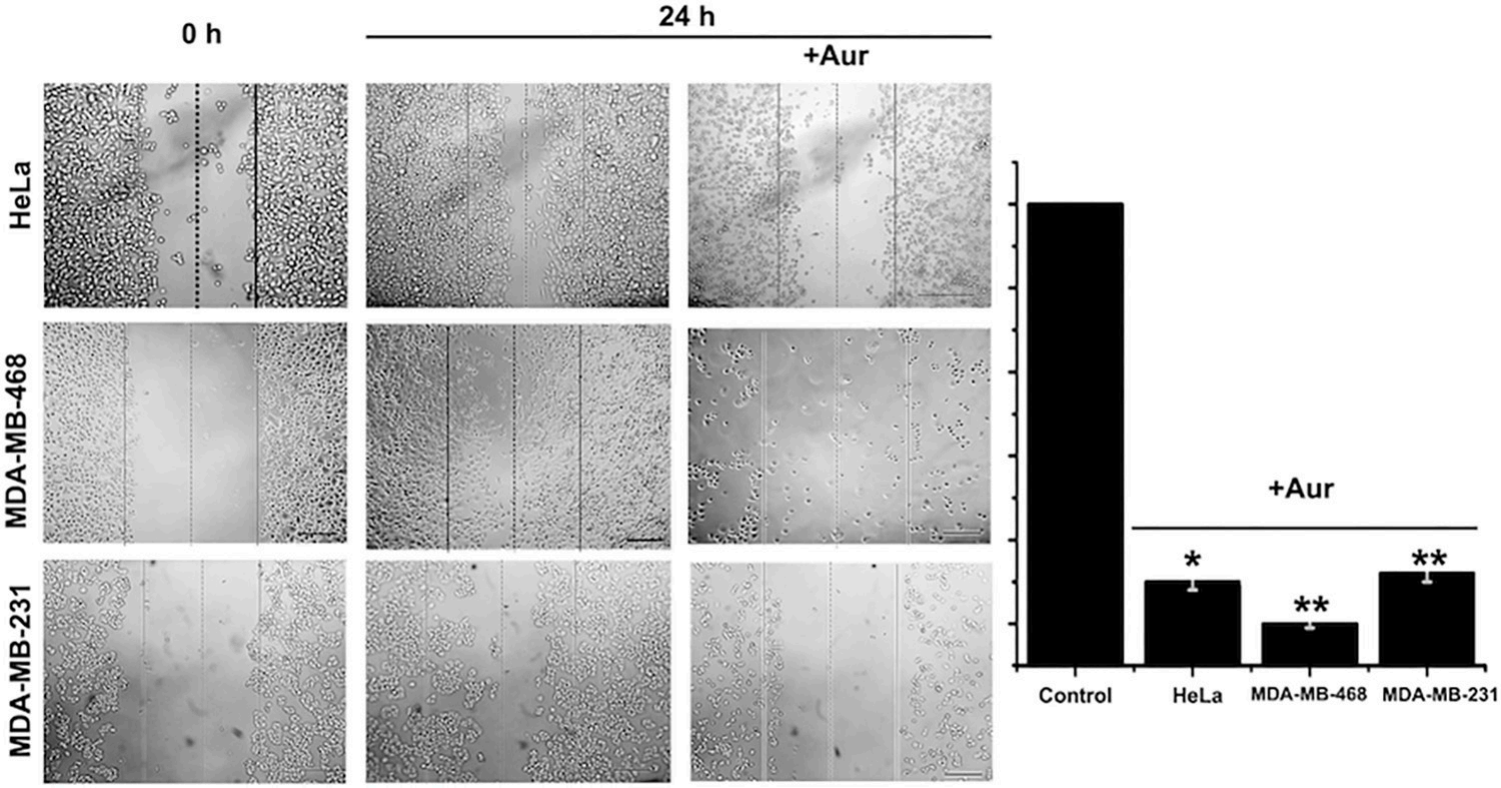
Migratory capacity in metastatic cancer cells exposed to IC_50_ Aur for 24 h. The Aur concentration added was 1.7, 0.8 and 2 μM for HeLa, MDA-MB-231 and MDA-MB-468 cells, respectively. n = 3; *p < 0.05, **p<0.01 *vs*. Control (Non-treated cells).

**Fig 5:**
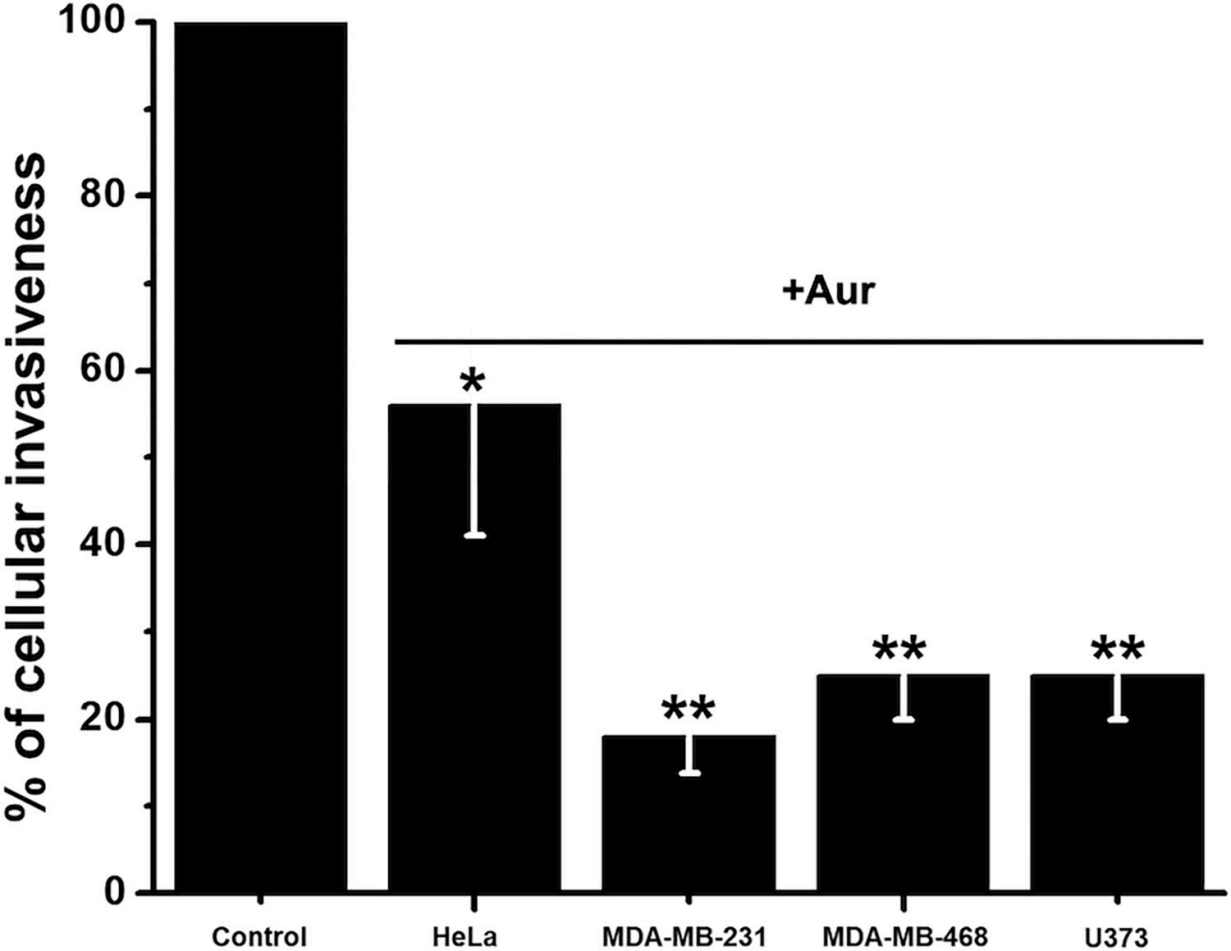
Cell invasiveness in metastatic cancer cells exposed to their IC_50_ Aur for 24 h. The Aur concentration added was 1.7, 0.8, 2 and 0.6 μM for HeLa, MDA-MB-231, MDA-MB-468 and U373 cells, respectively. n = 3; *p < 0.05, **p<0.01 vs. Control (Non-treated cells).

Metastasis seems to be a highly ATP-demanding process of malignant cancer cells [35]. Two of the processes involved in metastasis, cellular migration and invasiveness, were assessed for their sensitivity to Aur in the malignant human cervix (HeLa) and human TNBC (MDA-MB-231 and MDA-MB-468) cancer cell lines. Both metastatic functions were strongly inhibited by low Aur concentrations after 24 h incubation, although migration (90–95% inhibition; Fig 4) was indeed more sensitive than invasiveness (45–80% inhibition; Fig 5).

A similar inhibition of 75% on highly metastatic U373 glioblastoma cell invasiveness by 0.6 μM Aur was also attained (Fig 5). MA at 10 μM induced 65% inhibition on HeLa cell invasiveness (S1A Fig), whereas perhexiline at 4–20 μM prompted a 37–53% inhibition of HeLa, U373, HCT116, MDA-MB-231 and MDA-MB-468 cell invasiveness (S1B Fig). Migration of MDA-MB-231 and MDA-MB-468 cells was also 50–60% abolished by 15 μM perhexiline (S2 Fig). Again, it should be noted that dead cells cannot perform both migration and invasiveness, thus discarding unspecific drug effects on metastatic processes. Furthermore, after the 24 h exposure to drugs, cells are harvested, seeded and allowed to attach to the plate surface, which can only be performed by living cells.

### Aur and cisplatin synergically block metastatic cancer cell growth

To assess whether Aur may achieve greater inhibitory effects on cancer cell growth, Aur was combined with cisplatin (CP), one of the most widespread clinically used chemotherapy drugs for different cancer types, searching for inhibitory synergism. Encouragingly, supra-additive or synergistic effects of 19–52%, as revealed by the Bliss-type additivism (BTA) analysis [3], were attained for three highly metastatic cancer cell lines (Table 5). Combining Aur with other canonical chemotherapy drugs such as paclitaxel and doxorubicin to reveal further synergism is currently under experimentation.

**Table 5:** Synergistic inhibitory effect of Aur with CP on metastatic cancer cell proliferation.

Drug 1	assayed doses (μM)	Drug 2	assayed doses (μM)	C values (BTA %) [Range]	Experimental values (%) [Range]	Synergism (%) [Range]
**HeLa cells**						
CP	2–10	Aur	0.7–1.5	25 ± 10	77 ± 5.5	52 ± 11
				[16–36]	[71.5–82.5]	[39.5–61]
**U373 cells**						
CP	2–10	Aur	0.1–0.5	41 ± 7	69 ± 5	28 ± 4
				[34–47]	[64–74]	[23–30]
**MDA-MB-231 cells**						
CP	2–10	Aur	0.3–0.8	51 ± 8	70 ± 15	19 ± 7
				[41–56]	[53–83]	[11.5–26]

The indicated cancer cells (3–5 x 10^6^ cells) were cultured as described in Materials and Methods section for 24 h. Then, the indicated ranges of drug concentrations, which were just below the respective Aur IC_50_ values (Table 1), were simultaneously added, and cells were further cultured for additional 24 h. It is noted that the range of cisplatin (CP) concentrations used was well below (one or two orders of magnitude) its respective IC_50_ values. The Bliss-type additivism (BTA) was calculated as described in Material and Methods section. Drug synergism, *i*.*e*., supra-additive inhibitory effect on cell proliferation, was calculated from the difference between the experimental values and the BTA C values. The values attained for inhibition of cell proliferation at each particular drug concentration were pooled together for the indicated concentration ranges. The IC_50_ (μM) values of Aur and CP for cell proliferation were, respectively 1.7 ± 0.4 (6) and > 1000 (3) for HeLa cells; 0.6 ± 0.1 (3) and > 1000 (3) for U373 cells; and 0.8 ± 0.2 (3) and 72 ± 6 (3) for MDA-MB-231 cells.

The combination of Aur with CP also brought about a synergistic inhibition on the energy metabolism pathway fluxes, from 48% OxPhos flux inhibition by Aur alone (Table 2) to 59% by Aur + CP (Table 6), and from 75% glycolytic flux inhibition by Aur alone (Table 2) to 94% by the drug combination (Table 6). Previous analysis with CP alone at doses assayed in the present study (Table 5) showed no effect on proliferation rate, OxPhos, cellular invasiveness in HeLa cells [3].

**Table 6:** Synergistic inhibitory effect of Aur with cisplatin on HeLa energy metabolism.

	OxPhos		Glycolysis	
	nmol O_2_/min/mg protein		nmol lactate/min/mg protein	
	Control	+ Aura + CP*	Control	+ Aura + CP*
**HeLa**	4.8 ± 0.2 (3)	2 ± 0.6* (3)	16 ± 1 (3)	1 ± 0.5* (3)

The indicated drugs were incubated for 24 h and OxPhos and glycolysis fluxes of HeLa cells were determined as described under Material and Methods section and Table 2. As the oxygen consumption and lactate production measurements were made simultaneously with those shown in Table 2, the same control flux values were used for comparisons. Aur was added at 1.5 μM and cisplatin (CP) was 7 μM. *P < 0.05 *vs*. control (no added drugs).

## Discussion

A significant finding in the present study is the identification of synergistic combinations of repurposed drugs such as auranofin and meclofenamic acid with canonical anticancer drugs like cisplatin, which appears clinically relevant for improving current treatments. Aur seems a particularly promising redox-modulating molecule that warrants investigating further its mechanisms of action.

Although it has been demonstrated that Aur is unstable in aqueous media [50, 51], the effect of Aur on several cellular processes such as inhibiting antioxidant system, arresting cell cycle, inducing cellular death [52–54], or affecting energy pathways (this study) is clearly observed after 24 h treatment in several metastatic cancer cells and cancer stem cells, demonstrating that despite its instability, Aur is evidently effective. Thus, the present results correlated with the observation in patients receiving oral Aur, that high blood Aur levels (49–310 ng/mL; 0.1–0.6 μM) are found after 24 h -7 d of treatment [55, 56]. Therefore, the Aur instability in water could be attenuated by its interaction with proteins and cells or alternatively, Aur decomposing derivatives are the active drugs. This clearly deserves further research.

Indeed, Aur effective doses to decrease cancer cell proliferation were in the low micromolar range. This Aur concentration range was also effective to inhibit mitochondrial metabolism and glycolysis. Thus, Aur may be considered as a promising potent and specific anti-cancer drug by mainly affecting the energy metabolism pathways, without apparently perturbing non-cancer cells at the low doses used in the present study.

However, some limitations of the present study must be recognized. The non-cancer cells used in the present study, 3T3 fibroblasts and H9C2 cardiomyocytes, might not be the most rigorous control cells for comparisons with cancer cells. Although fibroblasts proliferate at a faster rate than other non-cancer cells [57], they do not proceed from the tissues of origin of the cancer cells used in the present study.

Another limitation is that the analysis of the drug effects was carried out at only one lengthy incubation-time of 24 h. This experimental design might involve a confounding effect since all observed effects could be related to cell death rather than to a drug specific action. Further experimentation at shorter times is required to better distinguish drug toxic specific effects. However, cells were washed out after drug exposure and the ensuing function assays were performed with living cells (with high viability) and isolated mitochondria. For a more accurate mechanistic understanding of the drug actions, it would be required to determine cell functions at several different shorter exposure times, although the short-term experiments with isolated mitochondria clearly demonstrated a direct drug effect on OxPhos and the long-term experiments with cells demonstrated that mitophagy was stimulated and migration and invasiveness, two ATP-dependent metastatic processes, were indeed inhibited. Then, it seems that cell death was prompted by drug-induced OxPhos inhibition.

### Aur and MA inhibit cancer growth and block OxPhos in cancer cells and isolated mitochondria

Aur exhibits a relevant anti-proliferation activity in bidimensional and *in vivo* models of several types of human cancer cell lines [53, 58, 59]. Indeed, Aur IC_50_ values reported in the present study were in the same range reported for other human cancer (hepatoma Hep3B, Calu‑6 and A549 lung) cells [58, 59]. The MA IC_50_ proliferation values determined here were in the range reported for small cell lung carcinoma cells [26] and for different NSAID-metal complexes in several metastatic and low-metastatic cancer cells [60]. It has been described that apoptosis induced by Aur in combination with glutaminase inhibitor CB-839 is one of the mechanisms associated to Aur proliferation blocking [20].

Several targets of Aur have been described, although it is thought that its primary mechanism of action is disrupting the redox homeostasis by inhibiting cytosolic and mitochondrial TrxRs and in consequence acting as a pro-oxidant agent. Here, energy metabolism was identified as another primary Aur target, as OxPhos flux was significantly abolished by Aur in cancer cells and isolated mitochondria. In fact, this is the first study showing a direct inhibitory effect of Aur at the mitochondrial level.

Aur and MA exhibited direct uncoupling and inhibitory effects on cancer and non-cancer isolated mitochondria. Similar effects were previously described for other NSAIDs such as diclofenac, piroxicam, indomethacin, nimesulide, meloxicam [61] and celecoxib [4, 5]. Inhibition of oxygen consumption in intact cells and state 3 (ADP-stimulated) respiration in isolated mitochondria by NSAIDs is linked to inhibition of the ATP/ADP translocator, ATP synthase and respiratory complexes I and II [4, 5, 61].

On the other hand, the stimulatory effect on basal respiration (uncoupling effect) by NSAIDs was by inducing collapse of the electrical membrane potential. The collapse of the electrical membrane potential, and the ensuing increased respiratory rate, may be due to either an increase in the conductance of cations towards the mitochondrial matrix or an increase in the activity of the respiratory complexes. Then, it may appear to be a confounding effect to observe NSAID inhibition of oxygen consumption in intact living cells. However, it should be noted that NSAIDs also inhibited oxygen consumption associated to ATP synthesis (*i*.*e*., oxidative phosphorylation, OxPhos) or state 3 (ADP-stimulated) respiration in isolated mitochondria. Basal respiration in isolated mitochondria is determined in the absence of adenine nucleotides added, which is not a physiological condition. Then, assessing oxygen consumption rates in living cells, which are able to homeostatically maintain their respective functions, for instance maintaining stable the adenine nucleotide concentrations at physiological levels for cellular work (*i*.*e*., ATP hydrolysis) and synthesis (*i*.*e*., OxPhos), resembles a near state 3 condition, *i*.*e*. cells require to continuously synthesize ATP as long as they stay alive.

In this regard, it has been described [62] that Aur decreased the mRNA and protein contents of respiratory chain (RC) complexes I, III, and IV, as well as complex V (ATP synthase) in ovarian cancer A2780 cells. The lower RC complexes content correlated with a slight inhibition (around 25%) of cancer cell total oxygen consumption, but the actual OxPhos flux, by titrating oxygen consumption with oligomycin, an ATP synthase inhibitor [35], was not examined. Determination of the oligomycin-sensitive respiration is necessary to correctly asses OxPhos flux, because there are several oxygen-consuming enzymes not coupled to mitochondrial ATP synthesis, which are overexpressed in cancer cells [35] and which may be also sensitive to Aur.

Aur also affected cancer glycolysis. This observation was opposite to that found in ovarian cancer A2780 cells, where glycolysis was significantly stimulated by Aur, presumably in response to the OxPhos impairment [62]. However, lactate produced by glutaminolysis and glycogenolysis was not accounted for to reveal the true glycolysis flux [35]. On the contrary, our observations correlated with studies [20] where several glycolysis proteins (*i*.*e*., GAPDH, PGAM, TPI, ALDH and ALDO) were significantly oxidized by Aur treatment (2.5 μM/ 8 h) in OVCAR-8 cells. The most oxidized enzyme induced by Aur was GAPDH, which in turn, showed a 40% lower activity *vs*. non-treated cells [20]. Unfortunately, glycolysis flux was not determined in Aur-treated OVCAR-8 cells for comparative purposes.

MA treatment of small cell lung carcinoma DMS114 cells caused downregulation of glycolytic proteins (ALDOA, GAPDH, ENO1, PyKM2 and LDHA) as well as upregulation of OxPhos proteins (cytochrome b-c1 complex subunit 2 and cytochrome c oxidase subunit 5B) [26]. In the present study, OxPhos flux inhibition by MA was observed. Therefore, MA also affects energy metabolism of cancer cells.

### Aur promotes mitophagy but not ROS production

An active mitophagy was observed as result of OxPhos impairment induced by Aur. The formation of autophagic vesicles induced by Aur has been also observed in human retinal pigment epithelial ARPE-19 cells treated with Aur (4 μM/ 4 h and 24 h) [63].

It may be argued that the energy metabolism inhibition by Aur derived from its effect on TrxR inducing a generalized oxidative stress or through an active mitophagy. However, the intracellular content and production of ROS was *diminished* in HeLa cells treated with Aur. Aur promotes activation of NRF2, a key transcriptional factor that upregulates enzymes involved in antioxidant metabolism such as TrxR, sulfiredoxin, glutamate cysteine ligase, glutathione S-transferase, and glucose-6 phosphate dehydrogenase in ovarian cancer A2780 cells. The GSH levels were also increased with Aur [62]. Then, these other observations support our present findings that after 24 h treatment, Aur promotes an activation of the antioxidant system decreasing the intracellular ROS produced by impaired OxPhos.

### Aur inhibits metastasis in cancer cells

Another significant finding of our study was that metastasis of HeLa, TNBC and U373 cells was markedly inhibited by Aur. Similar results were observed in human osteosarcoma KHOS, KRIB and BTK143 cells injected intrafemorally, which were able to establish and grow in the host but their metastatic potential was profoundly abolished by Aur (1μM/24 h) [64].

### Aur synergism with cisplatin blocks OxPhos

To prevent the harmful side effects induced by anti-cancer drugs, novel chemotherapy strategies based on the simultaneous use of two or more drugs might achieve greater efficacy, but given at comparatively lower doses. The aim is to target two or more different essential cancer cell processes such as cell proliferation, signaling, and/or metabolism. In this regard, the repurposing of approved drugs could be a promising alternative anti-cancer strategy. The rationale is that the careful selection of such drugs may have advantages in that (i) they should show fewer side effects; (ii) they may have unconventional but effective targets in tumor cells; and (iii) their use may decrease the overall cost and time associated with the development of brand-new chemotherapeutics [65, 66].

Aur and MA may be used for the treatment of pain, fever, inflammation, and rheumatic diseases [9, 24]. However, the combination of celecoxib, another repurposed NSAID with potent anticancer activity, with temozolomide, cisplatin, gemcitabine, or etoposide, has yielded rather modest responses on tumor growth, with side effects including dyspepsia, diarrhea, and abdominal pain [67, 68].

In contrast, studies with Aur in combination therapies have shown encouraging results. Aur at 2 μM and an inhibitor of the Wnt/β-catenin pathway synergistically depressed growth and invasion of human colon cancer cells, growth of subcutaneous xenograft mice models and restrained metastasis in lung metastasis mice models by inhibiting the phosphorylation of STAT3 and inducing caspase-3-dependent apoptosis [69]. However, assessment of this drug combination was not undertaken in non-cancer cells. Moreover, combination of Aur with anti-PD-L1 monoclonal antibody synergistically impaired the growth of 4T1.2 primary tumor [13].

Here we showed that besides their anti-inflammatory activity, Aur and MA show anticancer effects at micromolar doses, at least partially mediated by their effects on mitochondrial metabolism. This Aur mitochondrial impairment was synergistically enhanced by CP. The results from our study are consistent with those published by other groups. Liu et al, (2019) [70] demonstrated that Aur (0.25 μM) + CP (1 μM) for 72 h induced mitochondrial dysfunction and DNA damage in small lung cancer cells [70]. Moreover, Aur (1 μM) plus celecoxib (10 μM) for 24 h induced a decrease (> 30%) in both energy pathways in colon DLD-1, HCT116 and HT-29 cancer cells [71]. However, glycolysis was determined as extra cellular acidification rate which it is not the most appropriate approach for glycolysis determination [35].

Furthermore, in preliminary studies, the effect of Aur (0.7–1.5 μM) plus MA (2–4 μM) combination was assayed on HeLa cells growth at sub IC_50_ doses. Bliss type additivism analysis revealed a synergism value of -35% indicating a strong infra-additive effect in which both drugs exerted a null response on cellular growth [3]. Thus, this drug combination may not be a promissory alternative against cervix cancer. Further research is required to elucidate the molecular mechanisms explaining why MA decreases the Aur toxicity in cervix cancer and to test the combination drug effect on other metastatic cancer cells (Table 1).

In conclusion, the present findings may provide guidance for improving current clinical protocol treatments by using multi-target drugs or drug combinations based on auranofin.

## Supporting information

S1 Fig Cancer invasiveness assay in metastatic cancer cells exposed to MA (A) and perhexiline (B) for 24 h. All cancer cell invasiveness was compared with MDA-MB-231 cells, which are cancer cells with the highest invasiveness ability [4]. n = 3; *P < 0.05 vs. MDA-MB-231 non-treated cells.
(DOCX)

S2 Fig Migratory capacity in metastatic triple negative breast cancer cells exposed to perhexiline. n = 3; *P < 0.05 vs. Control (Non-treated cells).
(DOCX)

S1 Table Effect of several drugs on metastatic, low metastatic cancer and non-cancer cells growth. (DOCX)

S2 Table Therapeutic Index ratio (TI ratio) for Auranofin (Aur) and Meclofenamic (MA) acid in bi-dimensional mouse 3T3 fibroblast or mouse H9C2 cardiomyocytes *versus* cancer cells. (DOCX)

S3 Table Effect of Aur on oxygen consumption rates of cancer and non-cancer isolated mitochondria with succinate as oxidizable substrate. (DOCX)

## Abbreviations

Aur: auranofin
CP: cisplatin
GSH: reduced glutathione
IC_50_: concentration to attain 50% drug inhibition
MA: meclofenamic acid
NSAIDs: non-steroidal anti-inflammatory drugs
OxPhos: oxidative phosphorylation
TNBC: triple negative breast cancer
