# Supplementary material for: Repurposing auranofin and meclofenamic acid as energy-metabolism inhibitors and anti-cancer drugs
Source: PLoS One. 2024 Sep 17;19(9):e0309331. doi: 10.1371/journal.pone.0309331 (PMC11407620; doi:10.1371/journal.pone.0309331)
Supplement: S1 Fig — Cancer invasiveness assay in metastatic cancer cells exposed to MA (A) and perhexiline (B) for 24 h. All cancer cell invasiveness was compared with MDA-MB-231 cells, which are cancer cells with the highest invasiveness ability [4]. n = 3; *P < 0.05 vs. MDA-MB-231 non-treated cells. (DOCX) [file pone.0309331.s001.docx]

**Supplementary material**

**S1 Figure**

**
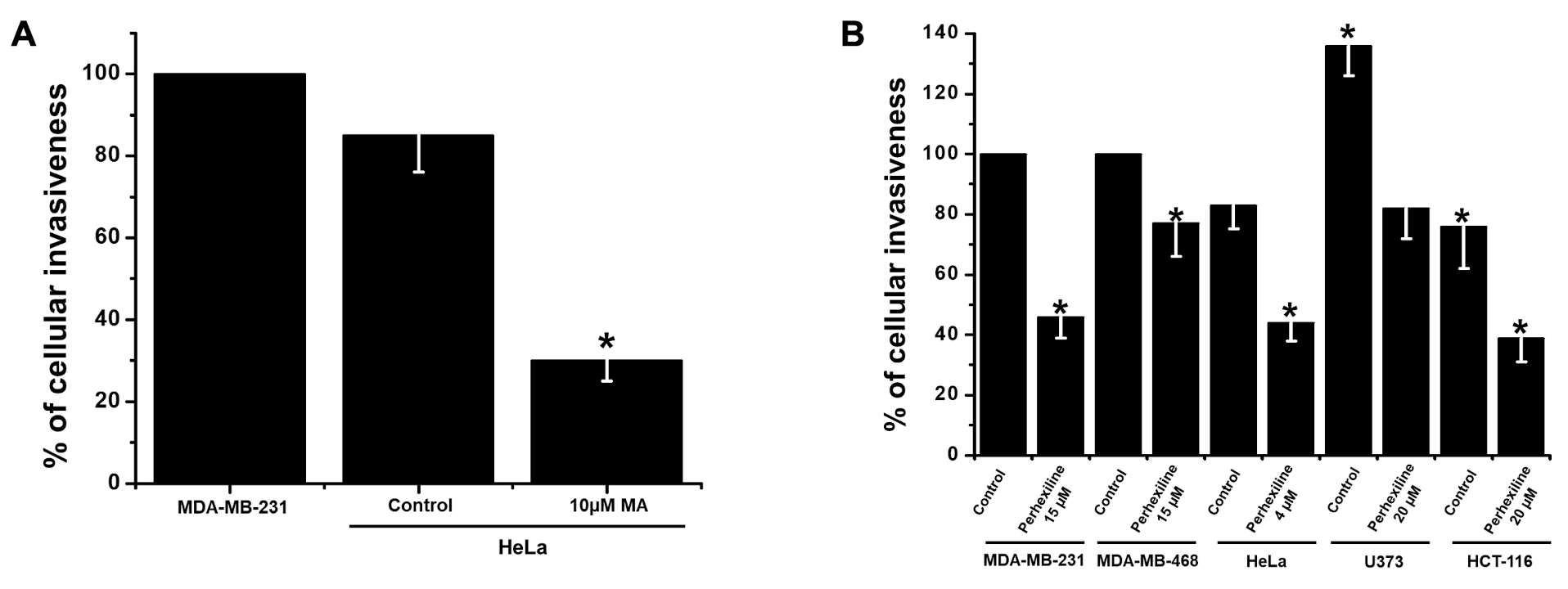
**

**
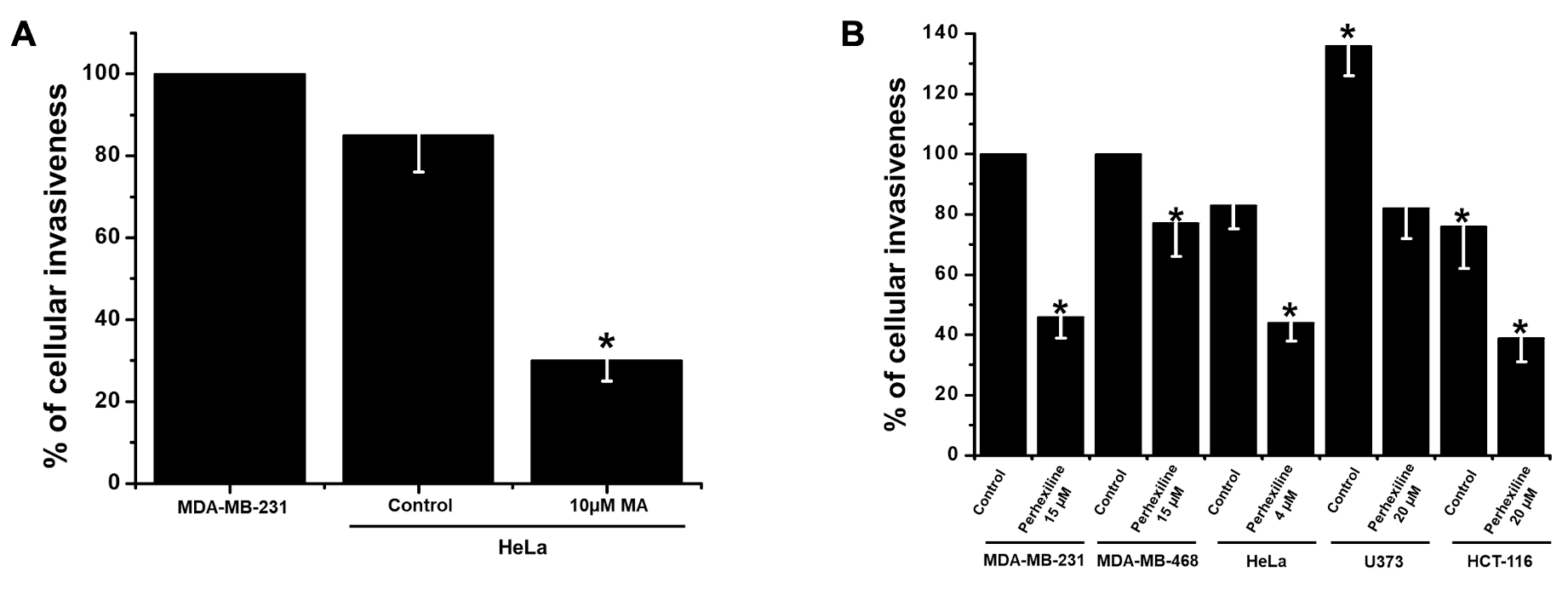
**

**S1 Figure**. Cancer invasiveness assay in metastatic cancer cells exposed to MA (A) and perhexiline (B) for 24 h. All cancer cell invasiveness was compared with MDA-MB-231 cells, which are cancer cells with the highest invasiveness ability [4]. n=3; *P < 0.05 *vs*. MDA-MB-231 non-treated cells.
