# Supplementary material for: Repurposing auranofin and meclofenamic acid as energy-metabolism inhibitors and anti-cancer drugs
Source: PLoS One. 2024 Sep 17;19(9):e0309331. doi: 10.1371/journal.pone.0309331 (PMC11407620; doi:10.1371/journal.pone.0309331)
Supplement: S2 Fig — n = 3; *P < 0.05 vs. Control (Non-treated cells). (DOCX) [file pone.0309331.s002.docx]

**Supplementary material**

**S2 Figure**

**
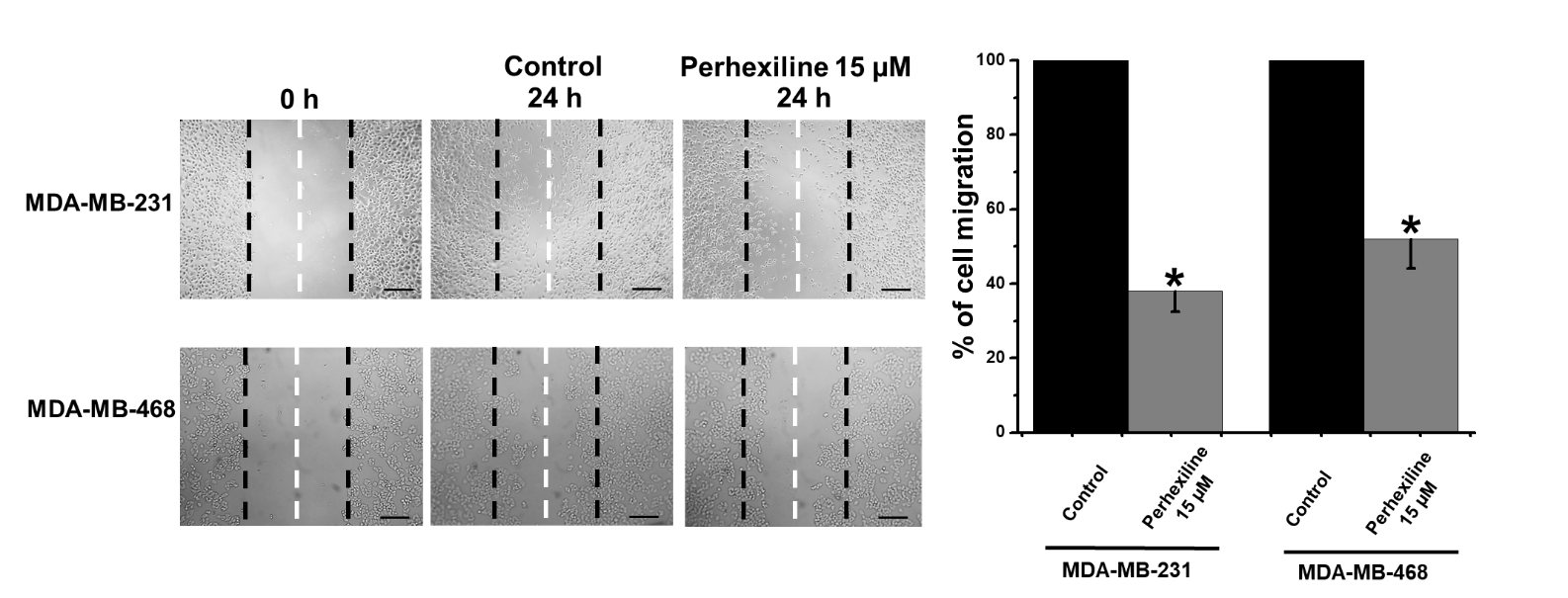
**

**S2 Figure.** Migratory capacity in metastatic triple negative breast cancer cells exposed to perhexiline. n=3; *P < 0.05 *vs*. Control (Non-treated cells)
