# Supplementary material for: Repurposing auranofin and meclofenamic acid as energy-metabolism inhibitors and anti-cancer drugs
Source: PLoS One. 2024 Sep 17;19(9):e0309331. doi: 10.1371/journal.pone.0309331 (PMC11407620; doi:10.1371/journal.pone.0309331)
Supplement: S1 Table — (DOCX) [file pone.0309331.s003.docx]

**S1 Table**. Effect of several drugs on metastatic, low metastatic cancer and non-cancer cells

growth

| **IC_50_ (µM) for proliferation cell rate** | | | |
| --- | --- | --- | --- |
| Cell type | **Simvastatin** | **Perhexiline** | **Sulindac** |
| **Human metastatic cancer cells** | | | |
| Breast MDA-MB231 | 111± 45 (3) | 11±0.6 (4) | 463 (2) |
| Breast MDA-MB468 | N.D. | 15 ± 0.8 (4) |  |
| Cervix HeLa | 31±13 (4) | 4 ± 3 (10) | >500 (3) |
| Prostate PC3 | 31±16 (4) | N.D. | N.D. |
| Colorectal HCT116 | 147± 58 (3) | 16 ± 7 (3) | >500 (3) |
| Colorectal COLO205 |  | 16 ± 5 (3) | N.D. |
| Glioblastoma U373 | 131 ± 41 (3) | 18.5 ± 2 (3) | >500 (3) |
|  |  |  |  |
| **Human low metastatic cancer cells** | | | |
| Breast MCF-7 | 126 ± 65 (3) | 20.5 ± 10 (4) | >500 (3) |
| **Non cancer cells** | | | |
| 3T3 mouse fibroblasts | 222 ± 60 (3) | 81.5 ± 8 (6) | >500 (3) |
| H9C2 mouse cardiomyocytes | 100 ± 32 (3) | >100 (3) | >500 (3) |

Data shown represents the mean ± S.D. (*n*). N.D., not determined.
