# Supplementary material for: Repurposing auranofin and meclofenamic acid as energy-metabolism inhibitors and anti-cancer drugs
Source: PLoS One. 2024 Sep 17;19(9):e0309331. doi: 10.1371/journal.pone.0309331 (PMC11407620; doi:10.1371/journal.pone.0309331)
Supplement: S2 Table — (DOCX) [file pone.0309331.s004.docx]

**S2 Table.** Therapeutic Index Ratio (TI ratio) for Auranofin (Aur) and Meclofenamic (MA) acid in bi-dimensional mouse 3T3 fibroblast or mouse H9C2 cardiomyocytes *versus* cancer cells.

| **Chemotherapy drugs** | **TI ratio (3T3/ cancer cells)** | | | | | | | |
| --- | --- | --- | --- | --- | --- | --- | --- | --- |
|  | **MDA-MB-231** | **MDA-MB-468** | **HeLa** | **PC3** | **HCT-116** | **COLO 205** | **U373** | **MCF-7** |
| **Aur** | 52 | 21 | 25 | 6 | 14 | 7 | 70 | 8 |
| **MA** | 2 | ND | 17 | 17 | 1 | ND | ND | 1 |

|  | **TI ratio (H9C2/cancer cells)** | | | | | | | |
| --- | --- | --- | --- | --- | --- | --- | --- | --- |
| **Aur** | 97 | 39 | 46 | 12 | 26 | 13 | 130 | 15 |
| **MA** | 2 | ND | 17 | 17 | 1 | ND | ND | 1 |

TI ratios were calculated from the IC_50_ values of Aur or MA shown in Table 1. TI corresponds to the quotient of the IC_50_ values for 3T3 fibroblast or H9C2 cardiomyocytes divided by IC_50_ values for each cancer cell line. ND, not determined.
