# Supplementary material for: Repurposing auranofin and meclofenamic acid as energy-metabolism inhibitors and anti-cancer drugs
Source: PLoS One. 2024 Sep 17;19(9):e0309331. doi: 10.1371/journal.pone.0309331 (PMC11407620; doi:10.1371/journal.pone.0309331)
Supplement: S3 Table — (DOCX) [file pone.0309331.s005.docx]

**S3 Table**. Effect of Aur on oxygen consumption rates of cancer and non-cancer isolated mitochondria with succinate as oxidizable substrate

| **Hepatoma Mitochondria** | | | | | |
| --- | --- | --- | --- | --- | --- |
|  | 0 | 10 µM | 25 µM | 50 µM | 100 µM Aur |
| **Succinate 0.5 mM + Rotenone 2 µM** | | | | | |
| **Pseudo State 4** | 62 ± 25 | 80 ± 10 | 90 ± 31 | 83 ± 23 | 82 ± 34 |
| **State 3** | 115 ± 38 | 143 ± 18 | 147± 23 | 141.5± 65 | 104 ± 24 |
| **State 4** | 54 ± 18 | 73 ± 7.5 | 84 ± 15 | 70 ± 28* | 56.5 ± 7 |
| **Net State 3** | 53.5 ± 13 | 63 ± 13 | 56.5 ± 21 | 58.3 ± 63 | 26 ± 46 |
| **RC (State 3/ State 4)** | 2.1 ± 0.2 | 1.9 ± 0.5 | 1.7 ± 0.1 | 1.9 ± 0.3 | 1.8 ± 0.4 |
| **Rat Liver Mitochondria** | | | | | |
|  | 0 | 10 µM | 25 µM | 50 µM | 100 µM Aur |
| **Succinate 0.5 mM + Rotenone 2 µM** | | | | | |
| **Pseudo State 4** | 51 (2) | 33 (2) | 73 (2) | 63.5 (2) | 59 (2) |
| **State 3** | 125.5 (2) | 150 (2) | 161 (2) | 95.5 (2) | 64 (2) |
| **State 4** | 31 (2) | 68 (2) | 39.5 (2) | 40.5 (2) | 56 (2) |
| **Net State 3** | 75 (2) | 116 (2) | 88 (2) | 36.5 (2) | 20 (2) |
| **RC** | 4.6 (2) | 5.4 (2) | 4.8 (2) | 2.3 (2) | 2.5 (2) |

Isolated mitochondria (1mg protein/mL) were incubated in KME buffer (120 mM KCl, 20 mM MOPS, 1 mM EGTA) pH 7.20 plus 2 mM KH_2_PO_4_ and the indicated oxidizable substrates. For state 3 (ADP-stimulated) respiration, 300-600 nmol ADP were added. RC, respiratory control; n=3. To get the maximal reduction of molecular oxygen (zero oxygen concentration), sodium dithionite (Na_2_O_4_S_2_) was added at the end of each measurement.
